# Ameliorative effects of epigallocatechin-3-gallate nanoparticles on 2,4-dinitrochlorobenzene induced atopic dermatitis: A potential mechanism of inflammation-related necroptosis

**DOI:** 10.3389/fnut.2022.953646

**Published:** 2022-08-09

**Authors:** Mengguo Han, Xue Wang, Jian Wang, Dongcen Lang, Xiaohua Xia, Yongfang Jia, Ying Chen

**Affiliations:** College of Life Sciences, Henan Normal University, Xinxiang, Henan, China

**Keywords:** EGCG nanoparticles, atopic dermatitis, oxidative stress, Th1, Th2, necroptosis

## Abstract

Atopic dermatitis (AD) is a common autoimmune and chronic inflammatory cutaneous disease with a relapsing-remitting course. Necroptosis is a regulated necrotic cell death mediated by receptor-interacting protein 1 (RIP1), receptor-interacting protein 3 (RIP3), and mixed lineage kinase domain-like pseudokinase (MLKL), which is activated by tumor necrosis factor-α (TNF-α). However, the mechanism and the role of necroptosis have not been delineated in AD progression. (-)-Epigallocatechin-3-gallate (EGCG), the main biological activity of tea catechin, is well known for its beneficial effects in the treatment of skin diseases. Here, PEG-PLGA-EGCG nanoparticles (EGCG-NPs) were formulated to investigate the bioavailability of EGCG to rescue cellular injury following the inhibition of necroptosis after AD. 2,4-dinitrochlorobenzene (DNCB) was used to establish AD mouse models. As expected, topically applied EGCG-NPs elicited a significant amelioration of AD symptoms in skin lesions, including reductions in the ear and skin thickness, dermatitis score, and scratching behavior, which was accompanied by redox homeostasis restored early in the experiment. In addition, EGCG-NPs significantly decreased the expression of inflammatory cytokines like TNF-α, interferon-γ (IFN-γ), interleukin-4 (IL-4), interleukin-6 (IL-6), and interleukin-17A (IL-17A) in a time-dependent manner than those of in AD group. As a result, the overexpression of RIP1, RIP3, and MLKL in the entire epidermis layers was dramatically blocked by EGCG-NPs, as well as the expression ofphosphorylated p38 (p-p38), extracellular signal-regulated kinase 1 (ERK1), and extracellular signal-regulated kinase 2 (ERK2). These findings promote that EGCG-NPs formulation represents a promising drug-delivery strategy for the treatment of AD by maintaining the balance of Th1/Th2 inflammation response and targeting necroptosis.

## Introduction

Atopic dermatitis (AD) is one of the most common chronic inflammatory cutaneous diseases with a relapsing-remitting course characterized by pruritus, dry skin, and eczema lesions (1), and a prevalence of about 2–5% in adults and 10–20% in children worldwide (2). Even though the cause of AD is not completely understood, multiple etiologic factors appear to be involved in AD pathogenesis, including the combined interaction of genetic predisposition, immune dysregulation, skin barrier dysfunction, and environmental factors (1). Combined with skin barrier defects, the immune system provided the body’s first line of defense against environmental exacerbating factors-induced AD by activating non-specific and specific inflammatory responses. T-helper (Th) cells activation is the main step in AD pathogenesis occurring in different endotypes from different ethnic backgrounds, especially for the well-known Th1/Th2 imbalance (3, 4). More recently, Th1/Th2/Th17-associated molecules have been found to up-regulate at focal lesions in both acute and chronic AD (5).

Among all of the inflammatory cytokines, TNF-α has gained a lot of attention due to its multiple effects in promoting cells death, including receptor-interacting protein 1 (RIP1)-independent apoptosis, RIP1-dependent apoptosis (RDA), and necroptosis (6). Necroptosis, one of the precisely controlled inflammatory cell death pathways, is a form of programmed necrosis activated by RIP1-receptor-interacting protein 3 (RIP3)-mixed lineage kinase domain-like pseudokinase (MLKL) complex, which eventually induced cell membrane permeabilization and cell necrosis (7). What is more, TNF-α regulates necroptosis by activating the mitogen-activated protein kinases (MAPK) pathway, which mediates various biological processes including cell death, survival, growth, and differentiation (8). Ubiquitination of RIP1 activated MAPKs and NF-κB promoting cell survival and other non-death functions, while deubiquitinated of RIP1 induced the caspase-8-mediated apoptosis. In addition, excessive necroptosis has been reported to promote skin inflammation and severe cutaneous diseases by upregulation of RIP3, and phosphorylated MLKL. Unfortunately, the mechanism and function of necroptosis in AD are poorly understood (9–11).

As the main bioactive component of tea polyphenols, the health benefits of epigallocatechin-3-gallate (EGCG) have been widely used in treating a wide range of diseases, based on its anti-inflammatory and antioxidant properties (12). For example, EGCG has been used in treating sodium nitroprusside (SNP) and ultraviolet radiation b (UVB) induced skin damage and psoriasis by regulating inflammation, apoptosis, and oxidative stress (13). Currently, even though various degrees of side effects have been reported for long-term use, steroids, anti-histamines, and immunosuppressive agents have been regarded as the first-line therapy for the treatment of AD. Therefore, searching for more effective alternative therapeutic agents is a promising treatment direction for AD. Several studies have shown that natural bioactive products have the potential to treat AD against inflammation and oxidative stress, such as St. John’s wort, licorice, and bitter substances, as well as green tea extracts (14, 15).

However, the low bioavailability, instability, and cytotoxicity at a large dose limited the clinical application of EGCG. Encapsulated EGCG to create a formulation is an effective strategy to overcome the above disadvantages. For example, Scalia et al., prepared EGCG oil-in-water emulsion making EGCG permeation into deep region of stratum corneum (16). Furthermore, EGCG-loaded liposomes have been reported in wound-healing treatment (17). Poly (lactic-co-glycolic acid) (PLGA), approved by EMA (European Medicines Agency) and US FDA (Food and Drug Administration), is widely used in drug delivery system because of its attractive properties, such as safety, biocompatibility, biodegradability, and sustained release. Polyethylene glycol (PEG) can reduce immunogenicity and prolong biological half-time. Therefore, PEG-PLGA-based nanoparticles (NPs) have more powerful advantages, including increased water solubility, reduced aggregation, and improved stability (18). In the present study, PEG-PLGA-EGCG-NPs (EGCG-NPs) were prepared and the therapeutic effects of EGCG-NPs (75 mg/kg/day) against AD were evaluated by focusing on necroptosis, which will be helpful for a better understanding of the precise mechanisms in AD pathogenesis and for guiding the design of the therapeutic agents based on the necroptosis.

## Materials and methods

### Chemicals and reagents

Epigallocatechin-3-gallate (purity > 98%; Yuanye Biotechnology Co., Ltd, Shanghai, China) was dissolved in water (pH 3.0) and stored at −20°C. mPEG-PLGA (Poly lactiv-co-glycolic acid, 75:25, 45,000) was purchased from Daigang Biomaterial Co., Ltd (Jinan, China). 2,4-Dinitrochlorobenzene (DNCB) was purchased from Sigma (United States). Lactate dehydrogenase (LDH), glutathione (GSH), superoxide dismutase (SOD), total antioxidant capacity (T-AOC), and malondialdehyde (MDA) kits were obtained from Nanjing Jiancheng Bioengineering Institute (Nanjing, China). Hematoxylin and Eosin (HE) staining kit was purchased from Beyotime (Shanghai, China). Receptor interacting protein 3 (RIP3) and mixed lineage kinase domain-like pseudokinase (MLKL) were obtained from Proteintech Group, Inc. (Resemont, IL, United States). Tumor necrosis factor-α (TNF-α), interleukin-4 (IL-4), extracellular signal regulated kinase 1 (ERK1), extracellular signal regulated kinase 2 (ERK2) and receptor interacting protein 1 (RIP1) antibody were obtained from Boster Biological Technology (Pleasanton, CA, United States). Phosphorylated p38 (p-p38) antibody was obtained from Cell signaling (Shanghai, China). SP-9002 SPlink detection (Biotin-Streptavidin HRP detection system) and DAB kits (ZLI-9018) were purchased from Zhongshan Golden Bridge Bio-technology (Beijing, China). RNAiso Plus reagent was purchased from Takara (9108, Japan). EasyScript First-Strand cDNA Synthesis SuperMix was purchased from TransGen Biotech (Beijing, China). UltraSYBR Mixture (with Rox I) was purchased from Cwbio (Beijing, China). All other reagents were of analytical grade.

### Preparation of epigallocatechin-3-gallate nanoparticles

Epigallocatechin-3-gallate nanoparticles were prepared by the double emulsion method in three steps: (1) The 18 mg PEG-PLGA polymer was dissolved in 1.5 ml of ethyl acetate into the oil phase (O). EGCG powder was dissolved in distilled water (pH 3.0) to make a 0.7% EGCG aqueous solution as an internal aqueous phase (W1). The W1/O emulsion was a mixture of oil phase and aqueous phase with a 1.5:1 ratio, which was sonicated with 20% amplitude for 20 s using an ultrasonicator; (2) Double emulsion (W1/O/W2) was obtained by adding W2 (2 ml) containing Tween 80 at 20% ultrasonic amplitude within 2 min. The entire procedure was carried out on ice; (3) After that, 0.02% Tween 80 (2 ml) was added, stirred, and evaporated on a magnetic stirrer for 24 h.

### Observation of the nanoparticles morphology with transmission electron microscopy

The morphology of the nanoparticles was studied by transmission electron microscopy (TEM) (JEM 1010, JEOL, Japan). The samples were negatively stained with 2% uranyl acetate before being placed on the surface of the grid for observation after the copper gate was activated by ultraviolet light. A drop of the sample was diluted at a ratio of 1:10 and operated at 25°C to observe whether the shape and size of the prepared nanoparticles were uniform, and whether there was aggregation and adhesion. The experiment was repeated three times.

### Measurement of size and zeta potential of nanoparticles

Using dynamic Light Scattering Particle size analyzer (Zetasizer Nano ZS; Malvern Instruments, Malvern, United Kingdom) measured the average particle size (Zav) and Zeta potential (ZP). A single drop diluted sample (1:10) was placed in the instrument and analyzed at 25°C. The experiment was repeated three times.

### Determination of the encapsulation efficiency

The concentration of EGCG was detected by using the Agilent 1100 HPLC system with C18 analytical column maintained at 40°C. The optimized mobile phase was constituted of acetonitrile and buffer. The flow rate was maintained at 0.8 ml/min, while the injection volume of the sample was set at 15 μl. The UV wavelength was fixed at 280 nm. Standards with a concentration range from 0.03 to 0.5 mg/ml were prepared. All experiments were done in triplicate. Then encapsulation efficiency was calculated using the following equation:


EE=(total⁢amount⁢of⁢EGCG-free⁢amount⁢of⁢EGCG)⁢/⁢total⁢amount⁢of⁢EGCG× 100%

### Animals

All animal procedures were performed in accordance with the Guide for the Care and Use of Laboratory Animals published by the China National Institute of Health and approved by the Animal Ethics Committee of Xinxiang Normal University. Twenty-seven Kunming mice (20–25 g) were purchased from Henan Kbeth Biotechnology Co., Ltd. All animals were required to undergo institutional quarantine for 7 days prior to use. The mice were housed in a temperature (23 ± 2°C) and humidity (55 ± 10%) enviro nment with a 12 h light/dark cycle, and provided with food and water *ad libitum*. A total of twenty-seven mice was divided into three groups (*n* = 9): (1) control group; (2) DNCB-treated group; (3) DNCB plus EGCG-NPs-treated group. The animals were sacrificed on day 7, day 14, and day 21 after AD, respectively. The EGCG treatment group was administered with EGCG-NPs at a dose of 75 mg/kg/day.

### Mouse atopic dermatitis model

**FIGURE 1:**
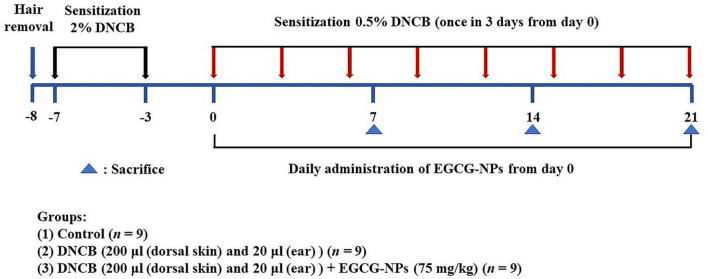
Experimental design and process of mice model.

To induce AD in mice, 1 day after dorsal hair removal (4 cm^2^), 200 and 20 μl of 2% DNCB in acetone/olive oil (3:1) were applied to the dorsal skin and ears for sensitization, respectively. In the challenge process, 200 and 20 μl of 0.5% DNCB were applied every 2 days for up to 21 days. Meanwhile, 75 mg/kg of EGCG-NPs was given daily to the ear and dorsal skin as the EGCG-NPs group. Mice were subjected to 1% pentobarbital general anesthesia (50 mg/kg intraperitoneal) on days 7, 14, and 21, respectively, and then cardiac blood and dorsal skin tissues were collected (Figure 1). Serum, dorsal skins, and ear samples were stored at −80°C. The others of dorsal skins and ear tissues were fixed in 4% paraformaldehyde for 48 h and then embedded in paraffin.

### Measure of skin lesion, dermatitis score, and ear thickness

The ear thickness was measured the day after each DNCB application by using an electronic vernier caliper (Decimal Caliper, Asa Dental spa, Lucca, Italy) and a digital camera to record clinical symptoms. The dermatitis score was evaluated the day after each DNCB application based on five symptoms: erythema/edema, dryness, erosion, excoriation, and lichenification, and each of the symptoms has 0 (none), 1 (mild), 2 (moderate), and 3 (severe) scores based on the skin lesion level (19).

### Scratching behavior

To investigate AD-like behavioral changes, the time of mice scratching the ear and dorsal skin in the hind paw for 30 min was measured after DBCB application, which was scored 0 (none), 2 (scratching shorter than 1.5 s), 4 (scratching longer than 1.5 s) (19, 20).

### Histological examination

For histological analysis, paraffin-embedded tissues were sectioned at 4 μm. Hematoxylin and eosin (H&E) staining was carried out to measure the changes in epidermal thickness. The stained tissues were observed and photographed by using optical microscopy (Leica, Wetzlar, Germany). The image was analyzed by using Image-J software.

### Superoxide dismutase, glutathione, malondialdehyde, total antioxidant capacity, and lactate dehydrogenase assays

The SOD, MDA, GSH, T-AOC, and LDH levels of dorsal skins and serum were detected by using the SOD, MDA, GSH, T-AOC, and LDH assay kit, respectively, according to the manufacturer’s instructions (Nanjing Institute of Jiancheng Biological Engineering, China).

### Immunohistochemical analysis

Deparaffinized sections were treated with 0.3% hydrogen peroxide in methanol for 15 min at room temperature to block endogenous peroxidase activity. The sections were incubated in 0.01 M, pH 6.5 sodium citrate buffer for 10 min at 121°C, and cooled to room temperature. After being blocked with 10% normal goat serum for 1 h at room temperature, the slides were subsequently incubated overnight with IL-4, TNF-α, p-p38, ERK1, ERK2, RIP1, RIP3, and MLKL. After being extensively washed with PBS, the slides were incubated with the Histostain-Plus kit. The sections were then counterstained with DAB. Quantitative evaluation was measured using Image-J software.

### Terminal deoxynucleotidyl transferase mediated dUTP nick-end labeling

Apoptotic cells were visualized by using the TUNEL assay kit. The fresh tissue was prepared into frozen sections of 10 μm, then detected with a TUNEL kit, stained with DAPI after operation according to the manufacturer’s instructions, and finally sealed with an anti-fluorescence quenching agent. After completion, all samples were observed using a fluorescence microscope and representative areas were captured.

### Quantitative RT-polymerase chain reaction analysis

According to the manufacturer’s instructions, total RNA was isolated from mouse dorsal skin using RNAiso Plus, and the extracted RNA was quantitatively analyzed by spectrophotometry. Complementary DNA (cDNA) synthesis was performed on the extracted RNA using the cDNA Synthesis Kit. Quantitative real-time polymerase chain reaction (QRT-PCR) was performed using synthetic cDNA and UL trasybr mixture (with Rox I). GAPDH mRNA was used as an internal control to normalize each targeted gene using the comparative 2^–Δ^ ^Δ^ *^Ct^* method. The primer sequences were listed as follows:

IFN-γ: forward 5′-TCAAGTGGCATAGATGTGGAAGAA-3′; reverse 5′-TGGCTCTGCAGGATTTTCATG-3′.

IL-6: forward 5′-TAGTCCTTCCTACCCCAATTTCC-3′; reverse 5′-TTGGTCCTTAGCCACTCCTTC-3′.

IL-17A: forward 5′-CCTCAAAGCTCAGCGTGTCC-3′; reverse 5′-GAGCTCACTTTTTGCGCCAAG-3′.

GAPDH: forward 5′-AGGTCGGTGTGAACGGATTTG-3′; reverse 5′-TGTAGACCATGTAGTTGAGGTCA-3′.

### Statistical analysis

All the data were expressed as the mean ± standard deviation (SD). The statistical analysis of the results was performed using GraphPad Prism 8.02 software (San Diego, CA, United States). One-way ANOVA followed by Tukey *post hoc* test was performed for group comparison. Values were shown as the mean ± standard deviation (SD). *n* = 3. *P* < 0.05 were considered to be a statistically significant difference.

## Result

### Physical characterization of epigallocatechin-3-gallate nanoparticles

**FIGURE 2:**
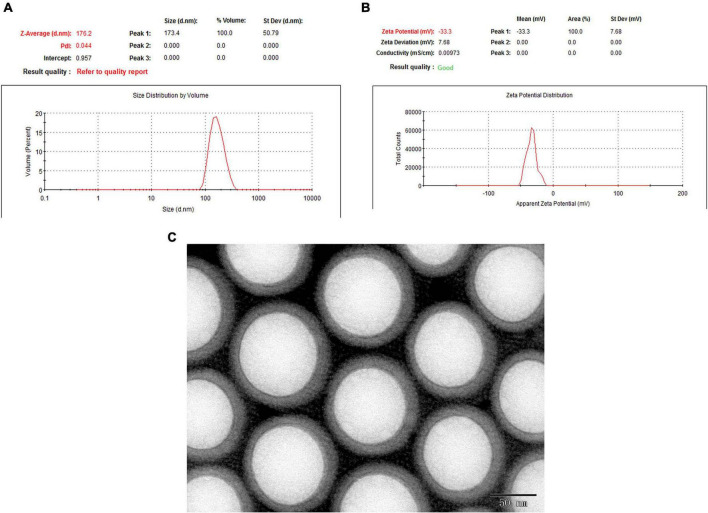
The morphology and the encapsulation efficiency of epigallocatechin-3-gallate nanoparticles (EGCG-NPs). **(A)** Size measurement and distribution of EGCG-NPs. **(B)** Zeta potential measurement of EGCG-NPs. **(C)** Transmission electron microscopy (TEM) image of EGCG loaded PLGA-PEG NPs (Scale bar = 50 nm).

Polyethylene glycol-PLGA-EGCG nanoparticles were formulated by using the double emulsion method and examined with dynamic light scattering, which showed 176.2 nm Zav, −33.3 mV ZP, and a lower PDI (0.044) (Figure 2). The morphology and the encapsulation efficiency of EGCG-NPs showed that the prepared nanoparticles had uniform size, no aggregation, no adhesion, regular arrangement, and approximate spherical shape with 86% encapsulation efficiency.

### Epigallocatechin-3-gallate nanoparticles alleviate the severity of skin lesion in atopic dermatitis induced by 2,4-dinitrochlorobenzene

**FIGURE 3:**
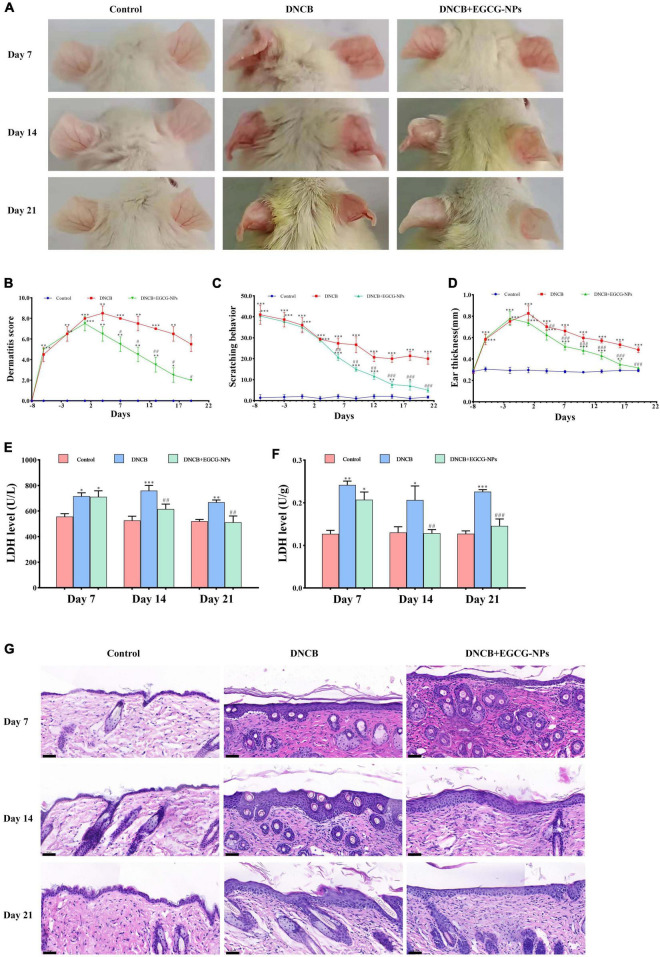
The effects of epigallocatechin-3-gallate nanoparticles (EGCG-NPs) on atopic dermatitis (AD) like symptoms and skin lesions induced by 2,4-dinitrochlorobenzene (DNCB). **(A)** The ears lesions of mice were photographed on day 7, 14, and 21. **(B)** Dermatitis score. **(C)** Scratching behavior. **(D)** Ear thickness. **(E)** LDH activity in serum. **(F)** Lactate dehydrogenase (LDH) activity in skin tissue. **(G)** The image of H&E staining in dorsal skin (200×, scale bar = 50 μm). *P < 0.05, ^**^P < 0.01, ^***^P < 0.001 vs. control. ^#^*P* < 0.05, ^##^*P* < 0.01, ^###^*P* < 0.001 vs. DNCB.

To investigate the severity of DNCB-induced AD, gross changes of the ear lesions were presented on days 7, 14, and 21 of the study, as well as AD symptoms, such as dermatitis score, scratching behavior, and ear thickness (Figures 3A–D). Compared to scratching behavior gradually decreasing over time, the trends of the dermatitis score and ear thickness were similar, which reached the peaks on day 4 and on day 1 after AD, respectively (*P* < 0.01 vs. Con, *P* < 0.001 vs. Con), and then gradually lowered in a time-dependent manner. Notably, EGCG-NPs significantly ameliorated clinical symptoms to the normal level, especially at the end of the experiment (*P* > 0.05 vs. Con), which was consistent with the changes in dorsal skin. Contrary to the normal stable epidermal thickness, DNCB dramatically induced epidermal hyperplasia 3.13-fold (*P* < 0.01) on day 7, and 4.10-fold (*P* < 0.001) on day 21, whereas treatment with EGCG-NPs significantly reduced hyperproliferation of lesioned epidermal cells (*P* > 0.05; Figure 3G and Table 1). Correspondingly, high levels of LDH in serum and tissue induced by DNCB were significantly inhibited in the EGCG-NPs group from day 14 of the experiment (*P* > 0.05 vs. Con) (Figures 3E,F). Taken together, EGCG -NPs alleviated the severity of skin lesions and the symptoms after AD.

**TABLE 1:** Measurement of epidermal thickness of mice dorsal skin after H&E staining.

	Control (μm)	DNCB (μm)	DNCB + EGCG-NPs (μm)
Day 7	21.90 ± 0.59	68.56 ± 2.61 (***)	50.08 ± 2.78 (***^###^)
Day 14	25.43 ± 0.45	123.85 ± 4.60 (***)	88.9 ± 1.28 (***^###^)
Day 21	26.83 ± 1.34	109.35 ± 2.87 (***)	43.56 ± 1.66 (^###^)

***P < 0.001 vs. control. ^###^*P* < 0.001 vs. DNCB.

### Epigallocatechin-3-gallate nanoparticles decreases oxidative stress of 2,4-dinitrochlorobenzene-induced atopic dermatitis

**FIGURE 4:**
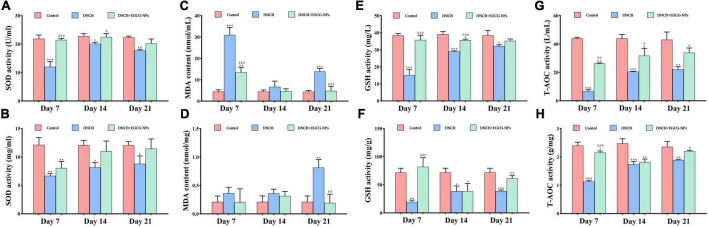
The effects of epigallocatechin-3-gallate nanoparticles (EGCG-NPs) on the expression of antioxidative activities after atopic dermatitis (AD). **(A)** Superoxide dismutase (SOD) activity in serum. **(B)** SOD activity in tissue. **(C)** malondialdehyde (MDA) content in serum. **(D)** MDA content in tissue. **(E)** Glutathione (GSH) activity in serum. **(F)** GSH activity in tissue. **(G)** T-AOC activity in serum. **(H)** T-AOC activity in tissue. *P < 0.05, ^**^P < 0.01, ^***^P < 0.001 vs. control. ^#^*P* < 0.05, ^##^*P* < 0.01, ^###^*P* < 0.001 vs. DNCB.

Altered redox-homeostasis has long been regarded as a plyer promoting AD development. Herein, antioxidant capacities were detected in both serum and tissue, such as SOD, MDA, GSH, and T-AOC (Figures 4A–H). Figure 4 showed that compared with the control group, EGCG-NPs maintained stable higher levels of SOD, GSH, and T-AOC in both serum and tissues than those in the AD group, especially on day 7 (*P* > 0.05). On the contrary, the serum and tissue MDA content after AD was significantly increased on day 7 (*P* < 0.001) and day 21 (*P* < 0.001), whereas EGCG-NPs inhibited the increase of MDA activity in serum and tissue to normal level at the end of the experiment (*P* > 0.05). Taken together, EGCG-NPs attenuated AD oxidative stress by elevating the activities of antioxidative enzymes.

### Epigallocatechin-3-gallate nanoparticles inhibit inflammatory response by improving Th1/Th2 imbalance after atopic dermatitis

**FIGURE 5:**
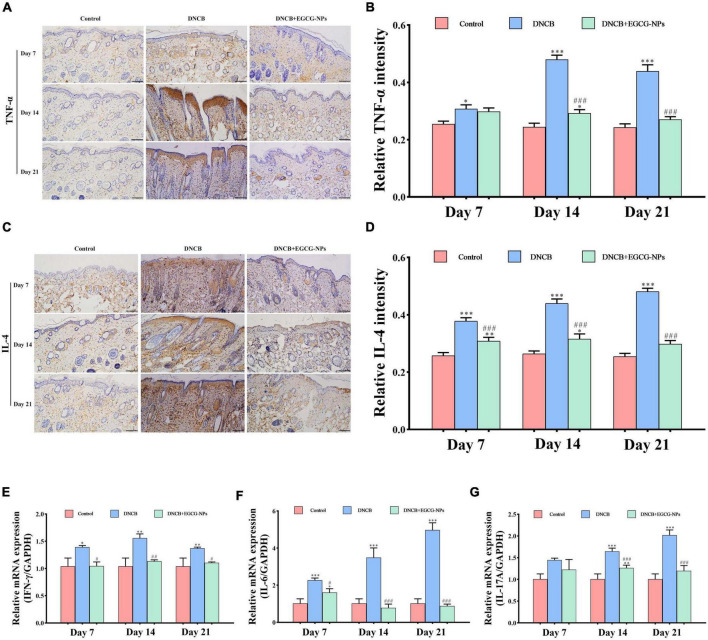
The effects of epigallocatechin-3-gallate nanoparticles (EGCG-NPs) on inflammatory cytokines of mice with atopic dermatitis (AD)-like skin lesions. Immunohistochemical microscope image of TNF-α **(A)** and IL-4 **(C)** in dorsal skin tissue (200×, scale = 100 μm). Quantification of TNF-α **(B)** and IL-4 **(D)** levels by densitometry. The mRNA expression level of IFN-γ **(E)**, IL-6 **(F)**, and IL-17A **(G)**. *P < 0.05, ^**^P < 0.01, ^***^P < 0.001 vs. control. ^#^*P* < 0.05, ^##^*P* < 0.01, ^###^*P* < 0.001 vs. DNCB.

To investigate the mechanism of EGCG-NPs in the treatment of AD, the expression levels of inflammatory cytokines Th1 (IFN-γ and TNF-α), Th2 (IL-4 and IL-6), and Th17 (IL-17A) were detected (Figure 5). The expression levels of IFN-γ, IL-6, IL-17A, TNF-α, and IL-4 after AD were significantly increased compared with the control group. Specifically, a faint staining signal of TNF-α was observed on day 7 after AD. In contrast, IL-4 positive signals were mainly located in the entire epidermis on day 7 after AD. With time, positive signals were observed in both the epidermis and dermis layers. Th1 cytokines TNF-α (*P* < 0.001) and IFN-γ (*P* < 0.01) peaked on day 14 but decreased on day 21 after AD, whereas Th2 cytokines (IL-4 and IL-6) and Th17 (IL-17A) persistently up-regulated and peaked on day 21 (*P* < 0.001). Importantly, the treatment of EGCG-NPs reduced the expression of all of these inflammatory cytokines in a time-dependent manner, especially at the end of the experiment (*P* > 0.05 vs. Con).

### Epigallocatechin-3-gallate nanoparticles inhibit the necroptosis rather than apoptosis in 2,4-dinitrochlorobenzene-induced atopic dermatitis

**FIGURE 6:**
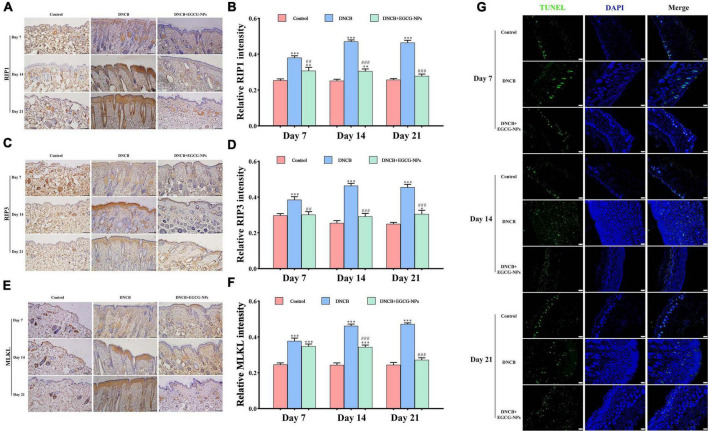
The effects of epigallocatechin-3-gallate nanoparticles (EGCG-NPs) on necroptosis in mice with 2,4-dinitrochlorobenzene (DNCB)-induced atopic dermatitis (AD). Immunohistochemical microscope image of RIP1 **(A)**, RIP3 **(C)**, and MLKL **(E)** in dorsal skin tissue (200×, scale = 100 μm). Quantification of RIP1 **(B)**, RIP3 **(D)**, and MLKL **(F)** levels by densitometry. TUNEL staining in dorsal skin tissue (100×, scale = 100 μm) **(G)**. *P < 0.05, ^**^P < 0.01, ^***^P < 0.001 vs. control. ^##^*P* < 0.01, ^###^*P* < 0.001 vs. DNCB.

To further explore whether inflammation triggers necroptosis after AD, the protein expression of RIP1, RIP3, and MLKL were measured (Figure 6). The overexpression of RIP1, RIP3, and MLKL was mainly observed in the entire epidermis after AD. It is worth noting that the maximum value of RIP1 (*P* < 0.001 vs. Con) and RIP3 (*P* < 0.001 vs. Con) reached on day 14 rather than that of MLKL (*P* < 0.001 vs. Con) on day 21. On the contrary, EGCG-NPs significantly inhibited the up-regulation of RIP1, RIP3, and MLKL in a time-dependent manner, especially on day 21 (*P* > 0.05 vs. Con). To further confirm that necroptosis rather than apoptosis predominantly occurred after AD, TUNEL was carried out. As expected, most of the fluorescence signals in the three groups were mainly observed in the dermis but not in the epidermis layer. Meanwhile, fewer apoptotic cells were induced by EGCG-NPs.

### Epigallocatechin-3-gallate nanoparticles suppressed the protein expression of mitogen-activated protein kinases signaling pathways after atopic dermatitis

**FIGURE 7:**
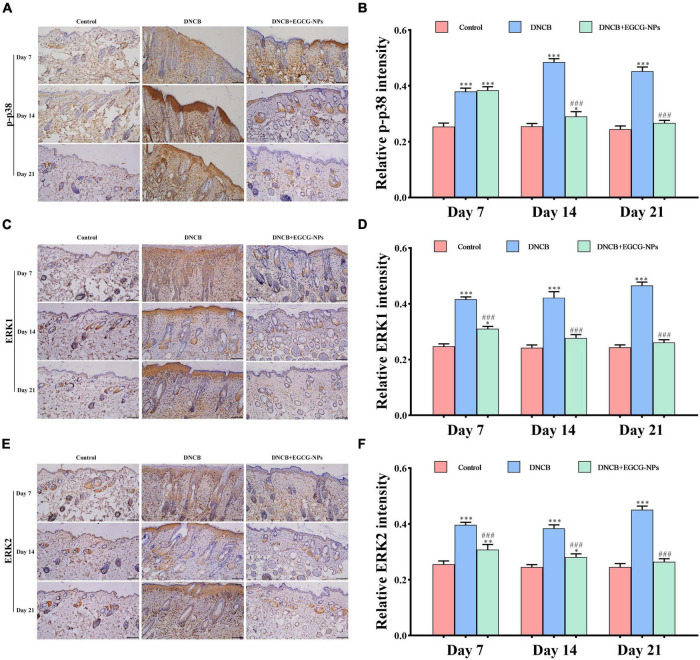
The effects of epigallocatechin-3-gallate nanoparticles (EGCG-NPs) on the expressions of mitogen-activated protein kinases (MAPK) signaling pathways in mice with 2,4-dinitrochlorobenzene (DNCB)-induced atopic dermatitis (AD). Immunohistochemical microscope image of p-p38 **(A)**, ERK1 **(C)**, and ERK2 **(E)** in dorsal skin tissue (200 ×, scale = 100 μm). Quantification of p-p38 **(B)**, ERK1 **(D)**, and ERK2 **(F)** levels by densitometry. *P < 0.05, ^**^P < 0.01, ^***^P < 0.001 vs. control. ^###^*P* < 0.001 vs. DNCB.

In order to explore the signaling pathways-mediated necroptosis after AD, the p-p38, ERK1, and ERK2 were detected by immunohistochemistry. As can be seen from Figure 7, contrary to consistently high expression of ERK1 and ERK2 throughout the experiment, p-p38 was mainly expressed on day 14 after AD. Meanwhile, activated MAPK pathway signals were located in both the epidermis and dermis layers. After treatment with EGCG-NPs, the over-expression of p-p38, ERK1, and ERK2 was restored to the normal level with time and mainly observed in the dermis layer. When quantified, the expression of p-p38, ERK1, and ERK2 exhibited a significant difference between EGCG-NPs and AD during the experiment, except for the expression of p-p38 on day 7. There were no significant differences between the EGCG-NPs and the control group on day 21, suggesting that EGCG-NPs alleviated AD by blocking MAPK signaling pathways.

## Discussion

Atopic dermatitis was the first-leading cause of skin diseases disability-adjusted life years (DALYs) in the world in 2017 (21). Although calcineurin inhibitors and/or topical corticosteroids are currently available, the local and systemic side effects associated with poor patient compliance and relapse cannot be ignored. As a result, optimized AD therapeutics with long-term safety and affordability will be highly needed. Recently, many natural products have exhibited similar potential pharmacological effects on AD treatment by targeting multiple signaling pathways (22). The health benefits of EGCG have been widely reported in various diseases as well as skin diseases, however, EGCG has failed in most, if not all, of clinical trials because of the limitations associated with the low stability and bioavailability (23). Recently, a nanoparticle formulation based on the biodegradable PLGA polymer was performed to enhance the bioactivities of EGCG in treating diabetes, wound healing, cancer, and neurodegenerative disease (24–26). In the present study, the EGCG-NPs were used as a novel therapeutic approach for the treatment of AD, including the following novel findings: (1) EGCG-NPs were successfully formulated; (2) EGCG-NPs decreased oxidative stress after AD; (3) EGCG-NPs attenuated the inflammatory response after AD; (4) EGCG-NPs weakened necroptosis by blocking the activation of MAPK signal, thereby improved the symptoms of AD lesions and alleviated the severity of AD.

Mounting evidence in laboratory and epidemiology has shown that EGCG exerts many beneficial health effects against UV irradiation, melanoma, psoriasis, as well as AD (27–30). However, some *in vivo* and *in vitro* pharmacological studies have demonstrated that EGCG at high concentrations and non-acidic conditions exhibited acute and subacute toxicity and prooxidative activities (31). Moreover, the hydrophobic features of EGCG made it difficult to penetrate into the deeper layers of the skin. Therefore, EGCG-NPs have been formulated and used in preclinical studies, which exhibited strong efficiency by inhibiting inflammatory response, skin barrier injury, and anti-aging, as well as antioxidative stress (16). In the present study, highly monodisperse uniform EGCG-NPs with a diameter of about 172.6 nm were successfully synthesized, which significantly alleviated the AD symptoms, including decreased dermatitis score, epidermal thickness, skin barrier injury, and the severity of skin lesions.

Oxidative stress is caused by the imbalance between ROS generation and antioxidant activity (32, 33), which has long been regarded as the main factor promoting AD development (34, 35). It has been found that higher levels of lipid peroxidation, reactive oxidative species (ROS), and lower levels of antioxidants contributed to eczema and acute exacerbation in AD patients (36–39). Therefore, many antioxidative agents have been used in treating AD by targeting oxidative stress, such as vitamin E, beta carotene, folic acid, and iron from diet and supplements (40). In the present study, EGCG-NPs significantly reduced systemic and local oxidative stress, eventually alleviating the symptoms of skin lesions after AD. Mounting evidence has reported that ROS and lipid peroxidation contributed to inflammatory skin diseases (41, 42). The immune changes in AD are characterized by the mixed immune response of Th1/Th2/Th17 (19, 43). Epidermal overexpression of IL-4, a well-known Th2 indicator, promoted almost all of the characteristics of AD, including pruritus, erosion, damaged skin barrier, and other symptoms (34, 44). Recently, dupilumab, an IL-4 receptor α monoclonal antibody, has been approved for the treatment of severe AD in adults by reducing the Th2 response and AD-associated epidermal abnormalities (45). Interestingly, EGCG-NPs can also inhibit the high expression of IL-4 mediated Th2 inflammatory response after AD, suggesting that EGCG-NPs can be a promising candidate in AD therapy. More importantly, the overexpression of IL-17A, TNF-α, and IFN-γ has also been inhibited by EGCG-NPs, indicating that EGCG-NPs exhibited a broad spectrum of anti-inflammatory activities.

More recently, more focus has been given to TNF-α mediated necroptosis, an alternative mode of regulated cell death, which contributed to neurodegenerative disease, cancer, and myocardial ischemia-reperfusion injury, as well as psoriasis (46–48). In the present study, necroptotic proteins mainly expressed and located in the epidermis, which is consistent with the expression of inflammatory cytokines. Normally, TNF-α binds to (tumor necrosis factor receptor 1) TNFR1 in the plasma membrane and sends a signal to RIP1, which in turn activates RIP3 to form the RIP1/RIP3 complex. Finally, phosphorylated MLKL translocated to the plasma membrane to induce perforation. Once damage-associated molecular patterns (DAMP) released into the extracellular environment from the membrane perforation, necroptosis might be triggered (49, 50). Additionally, oxidative stress has also been found to activate RIP1/RIP3/MLKL signaling pathway and enhance the formation of necrosome (51). Therefore, both ROS scavenger and inflammatory inhibitors have been used to inhibit necroptosis (52). However, RIP1, RIP3, and MLKL have been demonstrated in the inflammatory response by activation of inflammasomes either in a necroptosis-dependent manner or in a necroptosis-independent manner (53–55). Even though the causal relationship between necroptosis and inflammation is more complicated, the peaked expression of TNF-α and necroptotic proteins occurred simultaneously on day 14 after AD, suggesting that TNF-α might be an important trigger of AD necroptosis. The MAPK pathway, the downstream activation of oxidative stress, activated the inflammatory factor TNF-α, which is a key controller in the process of necroptosis via the modification of RIP1 and RIP3 (8, 56–59). Herein, EGCG-NPs significantly decreased the expression of ERK1/2 and p-p38 in the epidermis, indicating that EGCG-NPs regulated necroptosis after AD by oxidative stress/MAPK/inflammation pathway. The present study shows promising results. EGCG-NPs can be regarded as a new pharmacological agent because it restores necroptosis by inhibiting oxidative stress and inflammation. When treating AD either from a prevention or therapeutic standpoint, multiple signaling pathways should be focused on instead of examining each one alone, which might provide new approaches to the treatment of AD by targeting necroptosis, and will be helpful for guiding the design of the therapeutic agents.

## Conclusion

Results from the present study confirmed that EGCG-NPs formulation represents a promising drug-delivery strategy for the treatment of AD by restoring redox homeostasis and maintaining the balance of Th1/Th2 inflammation response and targeting necroptosis.

## Data availability statement

The original contributions presented in this study are included in the article/supplementary material, further inquiries can be directed to the corresponding author.

## Ethics statement

The animal study was reviewed and approved by China National Institute of Health and the Animal Ethics Committee of Xinxiang Normal University.

## Author contributions

YC, XX, and YJ designed the study. MH, XW, and JW performed the experiments. MH and XW performed data collection. MH, JW, and DL performed statistical analyses. YC and MH wrote the manuscript. All authors contributed to the article and approved the submitted version.
